# Parasitic diseases as a cause of acute abdominal pain: imaging findings

**DOI:** 10.1186/s13244-020-00892-5

**Published:** 2020-07-20

**Authors:** Emre Ünal, Sevtap Arslan, Mehmet Ruhi Onur, Erhan Akpinar

**Affiliations:** Department of Radiology, School of Medicine, Hacettepe University, 06100 Ankara, Turkey

**Keywords:** Abdominal parasitosis, Abdominal emergency, Imaging findings, CT, Acute abdomen

## Abstract

Acute abdominal pain can be seen in cases with parasitic diseases delivered to emergency departments. The diagnosis of the parasitic disease can be delayed because of the similar clinical signs encountered in other frequently seen causes of acute abdomen. Nevertheless, the features detected in imaging scans can be helpful in the diagnosis. The present study aims to raise awareness about abdominal parasitosis in emergency conditions and also to underline the association between imaging findings and the life cycle of parasites with illustrative cases.

## Key points

- Some types of parasites are endemic for certain locations.
- Immigration and travel are responsible for worldwide cases.
- A parasite may involve a specific organ or migrate through several organs.
- A parasite may induce a cyst formation that could be complicated with rupture, superinfection, or mass effect.
- Clinical and radiological findings may vary for the different types of parasites and their site of involvement.

## Background

Parasitic diseases are common. Although some types of parasites are endemic for certain locations, worldwide cases can be seen due to immigration and travel. A previously published systematic review reported that parasitic diseases resulted in 48.4 million cases annually and 59,724 deaths per year [1]. The diseases caused by most parasites results in significant morbidity and mortality among vulnerable populations. In the same study, researchers emphasize that enteric protozoa, ascariasis, and toxoplasmosis are the most common parasitic diseases; however, the global burden of disease is highest in cysticercosis. Therefore, these data indicate that there is no correlation between the incidence of the parasitic disease and the frequency and severity of symptoms [1]. Parasitic diseases may be transmitted in three different ways as (i) fecal-oral route, (ii) active penetration of the skin by larvae, and (iii) vector arthropods [2]. Infestation affects different parts of the body. Nevertheless, abdominal involvement is seen in the majority of cases. Although the clinical symptoms are usually nonspecific, patients may present with acute abdominal pain due to inflammatory changes in parenchymal organ, bowel walls, bile ducts, and peritoneal surfaces and obstructive changes in bowels and bile ducts. Acute abdominal pain may also result from complications of parasitic involvement such as abscess formation and rupture of focal parasitic cystic lesions. A parasite may be hosted by a specific intraabdominal organ, or may travel among several intraabdominal organs, or may induce a cyst formation that could be complicated with rupture, superinfection, or mass effect. Therefore, clinical and radiological findings may vary for the different types of parasites and also for their site of involvement. The present study aims to raise awareness about abdominal parasitosis that we encountered in our emergency radiology practice. A detailed literature search was also carried out to be able to summarize the epidemiologic aspects, radiological, clinical, and laboratory findings of these particular parasitic diseases (Tables 1 and 2) [3–19, 67, 74, 78].

**Table 1:** Epidemiologic aspects, route for transmission, and methods used for the definitive diagnosis of the parasitic diseases

Disease	Parasite	Epidemiology	Geographic distribution	^1^ Hosts/vectors ^2^ Transmission	Final diagnosis (routinely used method)
Cystic echinococcosis^(c)^ [3–6]	*E. granulosus*^(a)^	2–404/10,000	High prevalence in Mediterranean regions, southern and central parts of Russia, central Asia, China, Australia, South America and Africa	^1^ Definitive hosts (dogs and foxes) Intermediate hosts (sheep and human) ^2^ Ingestion of eggs	- Imaging based (primarily ultrasound findings) - Serologic assay - Aspiration of cyst content during imaging guided intervention
	*E. multilocularis*	0.2–3400/100,000	Asia, Central Europe and the northern parts of Europe, and North America		
	*E. oligarthrus*	Very rare, 106 human cases	Central and South America		
	*E. vogeli*				
Fascioliasis^(c)^ [3, 7]	*F. hepatica*^(a)^	0.9–6.1%	Worldwide, high prevalence in Europe and America	^1^ Intermediate hosts (Freshwater snails) Definitive hosts (herbivorous mammals, including humans) ^2^ Ingestion of watercress or contaminated water containing encysted larva	- Stool examination - Serologic assay
	*F. gigantica*		Tropical areas of Asia and Africa		
Ascariasis^(c)^ [3, 8]	*Ascaris lumbricoides*	8.8–22.3%	Asia, Africa, and South America	^1^ No intermediate host Humans are the only definitive hosts ^2^ Ingestion of fecally contaminated food	Stool examination
Toxocariasis^(c)^ [9]	*T. canis*^(a)^	0.7–15%	Worldwide	^1^ Intermediate hosts (rabbit, lamb, fowl) Definitive hosts (domestic dogs and cats). Humans are accidentally infected intermediate hosts ^2^ Ingestion of eggs from contaminated foods	Serologic assay
	*T. cati*				
Intestinal taeniasis^(c)^ [10–12]	*T. saginata*	0.7–4.9%	Sub-Saharan Africa and the Middle East, Eastern Europe, the Philippines, and Latin America	^1^ Intermediate hosts (pigs for *T*. *solium*, cattle for *T*. *saginata*) Definitive hosts (Humans are the only definitive hosts) ^2^ Ingestion of larvae for taeniasis and ingestion of eggs for cysticercosis	Stool examination
	*T. solium*		Worldwide; especially in Mexico, Latin America, West Africa, Russia, India, Manchuria, and Southeast Asia		
	*T. asiatica*		Taiwan, Korea, Indonesia, Nepal, Thailand and China		
Amoebiasis^(c)^ [13]	*E. histolytica*^(b)^	5–42%	Worldwide. Endemic in developing parts of Central and South America, Africa, and Asia	^1^ Humans are the principal host and reservoir ^2^ Ingestion of the cyst from fecally contaminated food or water	- Stool examination - The real-time PCR (to identify *E*. *histolytica*)
	*E. dispar*^(a)^				
	*E. moshkovskii*				
Malaria^(c)^ [14, 15]	*P. falciparum*^(b)^	5.2–75%	Tropical Africa, South America, South-eastern Asia, and Western Pacific	^1^ Vectors (*Anopheles* mosquitos) ^2^ Transmitted by infected female mosquitoes’ bites	Microscopic examination of blood
	*P. vivax*				
	*P. ovale*				
	*P. malariae*				
	*P. knowlesi*				
Visceral leishmaniasis^(c)^ [16]	*Leishmania*^(a,b)^*donovani*	200,000–400,000/per year	Northeast of the Indian subcontinent, East Africa	^1^ Vectors (*Phlebotomus* sandflies) ^2^ Transmitted by infected *Phlebotomus* sandflies’ bites	- Bone marrow sampling - Serological examination
	*Leishmania infantum*		Mediterranean region, Latin America		
Dientamoebiasis^(c)^ [17]	Dientamoeba fragilis	0.4–42%	Worldwide	^1^ Humans are the principal host ^2^ Fecal-oral (hypothetical transmission via *Enterobius vermicularis* eggs)	Stool examination
Anisakiasis^(c)^ [18]	Anisakis simple	3/1,000,000 in Japan	Japan, Korea, Latin America, and Europe (Scandinavia, The Netherlands, Spain, France, Britain). Over 90% of cases are from Japan	^1^ Intermediate hosts (different species of crustaceans, fish, or squid) Definitive hosts big sea mammals (whales, dolphins, or seals) Humans are accidentally infected intermediate hosts ^2^ Ingestion of raw or undercooked fish containing larvae of the anisakis worm	Endoscopy, histopathological examination

^a^Most common

^b^More severe clinical presentation

^c^References

**Table 2:** Parasitosis and affected abdominal organs. Associated radiological, clinical, and laboratory findings

Disease type		Imaging findings	Clinical presentation	Lab findings
Cystic echinococcosis^a^ [2, 3, 19–31]				
Liver	Perihepatic rupture	- Fluid collection through the course of cyst’s content - Mural thickening of the bowel loops and fat tissue stranding adjacent to cyst contents - Focal outward bulging, discontinuity of the cyst wall	RUQ or generalized abdominal pain, fever, nausea and vomiting, urticaria, and fatal anaphylaxis	Leukocytosis, elevated CRP (C reactive protein), eosinophilia, increase in serum aspartate transaminase (AST) and alanine aminotransferase (ALT) levels
	Biliary rupture	- Structural deformity, loss of spherical shape of the cyst - Dilatation of the intrahepatic bile ducts with linear filling defects within the biliary tract - Lipid-fluid level due to bile - Air or an air-fluid level within the cyst if superinfection is present	RUQ pain, jaundice, pruritus, fever, nausea and vomiting, anaphylactic reaction	Leukocytosis, elevated CRP, eosinophilia, elevated serum AST, ALT, gamma-glutamyl transferase (GGT), alkaline phosphatase (ALP), bilirubin, and amylase
	Vascular invasion	- Dilatation of the affected vein (most commonly hepatic veins) - Total occlusion or linear filling defects within the lumen - Parenchymal hemodynamic change at vascular territory	RUQ pain, dyspnea and chest pain in case of pulmonary embolism	Leukocytosis, eosinophilia, abnormal liver function tests and, hypoxemia and hypocapnia/hypercapnia in case of pulmonary embolism
	Biliary compression	- Dilatation of the intrahepatic bile ducts without linear filling defects; however, increased echogenicity/decreased T2 signal could be seen due to within the biliary tract. Dilatation of the intrahepatic bile ducts, enhancement of intrahepatic biliary duct, parenchymal changes (increased signal intensity on T2-weighted images, segmental parenchymal enhancement)	-RUQ, jaundice, pruritus -Fever, nausea and vomiting less common compared to biliary rupture	Eosinophilia, elevated ALP, GGT, and bilirubin
Kidney	Perirenal rupture	- Fluid collection through the course of cyst’s content - Focal outward bulging, discontinuity of the cyst wall	Flank pain, fever, nausea and vomiting, urticaria, and fatal anaphylaxis	Leukocytosis, elevated CRP, eosinophilia, high serum blood urea nitrogen (BUN), and creatinine
	Pelvicalyceal system rupture	- Structural deformity, loss of spherical shape of the cyst - Hydroureteronephrosis, pyonephrosis may accompany - Filling defects within the pelvicalyceal system - Urinoma may accompany in case of pelvicalyceal system rupture	Flank pain, fever, nausea and vomiting, urticaria, and fatal anaphylaxis	
	Pelvicalyceal system compression	- Hydroureteronephrosis without filling defects - Urinary dilatation is seen above the level of compression	Flank pain, fever, nausea and vomiting, urticaria, and fatal anaphylaxis	Eosinophilia, elevated BUN, and creatinine
Fascioliasis^a^ [2, 3, 32–43]				
Liver	Hepatic stage	- Multiple microabscesses, “tunnels and caves” sign - Subcapsular hemorrhage-effusion, focal liver capsule thickening and enhancement - Periportal lymphadenopathy, rarely portal vein thrombosis and wedge-shaped hemorrhagic infarction - Splenomegaly may accompany	RUQ pain, urticaria, right upper quadrant pain and fever, fatigue	Leukocytosis, eosinophilia, elevated liver enzymes
	Biliary stage	- Dilated biliary ducts, periportal thickening - Floating particles in the biliary system - Cholecystitis and/or pancreatitis may accompany	RUQ pain, pain could be disseminated in case of pancreatitis, jaundice, fever, nausea, diarrhea, pruritus	Leukocytosis, eosinophilia, elevated liver enzymes, hyperbilirubinemia, and elevated pancreatic enzymes in case of pancreatitis
Ascariasis^a^ [2, 3, 44–49]				
Gastrointestinal system (GIS)		-Tubular structure within the lumen of stomach or bowel, associated fat tissue stranding - Dilated bowels in case of intestinal obstruction - Rarely, intraabdominal free fluid, gas, and/or fluid collection in case of GIS perforation - Inflamed and distended appendix vermiformis	Abdominal distension, dyspepsia, nausea and vomiting in case of GIS obstruction, generalized abdominal pain fever and sepsis in case of GIS perforation, RLQ pain in case of acute appendicitis	Anemia, leukocytosis, eosinophilia
Liver		- Areas of decreased attenuation on CT, altered echogenicity on US due to parenchymal involvement - Filling defects within the biliary tree or gallbladder - Biliary dilatation, periportal inflammation - Gallbladder wall thickening and distention - Parenchymal or perihepatic abscess	RUQ pain, fatigue, fever, jaundice and pruritus in case of biliary dilatation, MURPHY sign positivity in case of acute cholecystitis, septic status can be seen in case of gallbladder perforation or liver abscess	Anemia, leukocytosis, eosinophilia, and elevated liver tests including bilirubin
Pancreas		- Thickening of pancreas, peripancreatic fat tissue stranding and fluid collection may accompany	Epigastric pain and acute pancreatitis can be the sole presentation or be seen following biliary involvement	Leukocytosis, eosinophilia, elevated pancreatic enzymes, mild hyperbilirubinemia, high ALP and GGT levels
Toxocariasis^a^ [2, 46–49]				
Liver		- Multiple, ill-defined, oval shaped, small lesions (< 2 cm) in parenchyma - Lesions may coalescence on follow-up - Splenomegaly may accompany	RUQ pain, fatigue, fever, nausea and vomiting	Leukocytosis, eosinophilia, mildly elevated liver enzymes
Taeniasis^a^ [2, 18, 50–56]				
Gastrointestinal system (GIS)		-Tubular structure within the lumen of stomach or bowel, associated fat tissue stranding - Dilated bowels in case of intestinal obstruction - Rarely, intraabdominal free fluid, gas and/or fluid collection in case of GIS perforation - Inflamed and distended appendix vermiformis	Abdominal distension, dyspepsia, nausea and vomiting in case of GIS obstruction, generalized abdominal pain fever and sepsis in case of GIS perforation, RLQ pain in case of acute appendicitis	Leukocytosis, eosinophilia, anemia
Liver		- Filling defects within the biliary tree or gallbladder - Biliary dilatation, periportal inflammation - Gallbladder wall thickening and distention	RUQ pain, fatigue, fever, jaundice and pruritus in case of biliary dilatation, MURPHY sign positivity in case of acute cholecystitis, septic status can be seen in case of gallbladder perforation	Leukocytosis, eosinophilia, hyperbilirubinemia and elevated liver enzymes
Pancreas		- Thickening of pancreas, peripancreatic fat tissue stranding and fluid collection may accompany	Epigastric pain and acute pancreatitis can be the sole presentation or be seen following biliary involvement	Leukocytosis, eosinophilia, elevated pancreatic and liver enzymes
Amoebiasis^a^ [44, 57–59]				
Colon		- Colonic wall thickening, mucosal edema and increased mucosal enhancement (primarily ascending colon) - Rarely, intraabdominal free fluid, gas and/or fluid collection in case of colonic perforation	Bloody diarrhea, tenesmus, fever, and abdominal pain. Generalized abdominal pain and sepsis in case of colonic perforation	Leukocytosis, anemia
Liver		- Parenchymal loculated fluid collection (abscess), air or an air-fluid level can be seen within the collection. - Perihepatic fluid and right-sided pleural effusion may accompany	RUQ pain, fever, colonic symptoms may accompany, sepsis in case of diffuse liver abscesses	Leukocytosis without eosinophilia, anemia, elevated liver enzymes, high erythrocyte sedimentation rate and CRP
Malaria^a^ [14, 60–66]				
Liver		- Hepatomegaly, periportal edema, perihepatic or intraabdominal ascites - Gallbladder wall thickening and distention due to acalculous cholecystitis	RUQ pain, fatigue and fever, MURPHY sign positivity in case of acute cholecystitis	Anemia, thrombocytopenia, leukocytosis, mild hyperbilirubinemia, mildly elevated liver enzymes
Spleen		- Areas of decreased attenuation on CT, altered echogenicity on US due to infarction, splenomegaly - Parenchymal or perisplenic hemorrhage/hematoma in case of spontaneous splenic rupture	LUQ pain Rapid clinical deterioration tachycardia and hemorrhagic shock in case of splenic rupture	
Gastrointestinal system (GIS)		- Dilated bowels in case of intestinal obstruction - Rarely, intraabdominal free fluid, gas, and/or fluid collection in case of GIS perforation	Nausea and vomiting in case of GIS obstruction, generalized abdominal pain fever and sepsis in case of GIS perforation	
Pancreas		- Thickening of pancreas, peripancreatic fat tissue stranding and fluid collection may accompany	Epigastric pain and symptoms related to acute pancreatitis	Anemia, thrombocytopenia, leukocytosis, hyperbilirubinemia, elevated liver and pancreatic enzymes
Visceral leishmaniasis^a^ [16, 67–72]				
Liver		- Hepatomegaly, ascites, right-sided pleural effusion - Nodular shaped focal parenchymal lesions	RUQ pain, fatigue and fever,	Pancytopenia, elevated CRP, hypergammaglobulinemia
Spleen		- Splenomegaly, ascites, left sided pleural effusion - Nodular shaped focal parenchymal lesions - Parenchymal or perisplenic hemorrhage/hematoma in case of spontaneous splenic rupture	LUQ pain, fatigue and fever, rapid clinical deterioration tachycardia, and hemorrhagic shock in case of splenic rupture	
Lymph nodes		- Enlarged and heterogeneous lymph nodes, central cystic changes can be seen in case of necrosis	Painful and palpable peripherally located lymphadenopathy	
Dientamoebiasis^a^ [17, 73]				
Gastrointestinal system (GIS)		- Bowel wall thickening, mucosal edema, associated intra-abdominal free fluid - Dilated bowels in case of intestinal obstruction	Abdominal pain, diarrhea, anorexia	Leukocytosis, rarely eosinophilia
Liver		- Periportal fat tissue stranding and/or edema	RUQ or epigastric pain, fever	
Anisakiasis^a^ [74–77]				
Gastrointestinal system (GIS)		- Severe submucosal edema of the involved gastrointestinal area, adjacent fat tissue stranding, enlargement of lymph nodes, intraabdominal free fluid may accompany. Rarely intestinal obstruction due to intussusception	Abdominal pain, nausea, vomiting, diarrhea, signs of peritoneal irritation Ileus/intestinal obstruction with or without intussusception	Leukocytosis

^a^References

## Hydatid cyst

Hydatid disease is a worldwide zoonosis produced by the larval stage of the *Echinococcus* tapeworm. There are 4 types of *Echinococcus* infections. *E*. *granulosu*s is the most common type, whereas *E*. *multilocularis* is less common but more invasive, mimicking malignancy [20]. *E*. *vogeli* and *E*. *oligarthrus* are very rare.

In humans, hydatid disease involves the liver in approximately 75% of the cases [21]. Although the liver is the most frequent site of involvement, any part of the body may host the hydatid disease. Hepatic hydatid cyst is commonly detected incidentally and patients are asymptomatic in most of the cases. Ultrasonography (US) can be used as a screening method of choice in liver hydatidosis. Gharbi classification system and World Health Organization (WHO) classification system classify the hydatid cysts based on their gray-scale ultrasound appearances. Unilocular or multilocular appearance, anechoic or echogenic content, multivesicular or multiseptated appearance, presence of hydatid sand, daughter cysts, floating membrane, and/or calcified wall are the sonographic features which determine the type of the hydatid cyst in Gharbi’s or WHO classification system [18, 22, 23]. Gharbi’s and WHO classification systems are helpful to determine to evaluate the cyst activity the appropriate treatment method (medical treatment, percutaneous drainage, or surgical excision) for the hydatid cyst type [22–25]. The sensitivity of computed tomography (CT) in liver hydatidosis is 94% [20]. Evaluation of cystic component, vascular and biliary tree involvement, and extrahepatic extension may be assessed with magnetic resonance imaging (MRI) [26].

**Fig 1.**
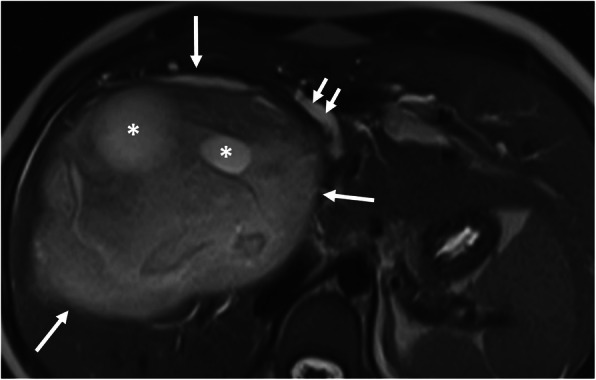
A 16-year-old girl was admitted to the emergency department with a 1-week history of pruritus, jaundice, and fever. Axial T2-weighted MR-image shows the imaging findings of intrabiliary rupture of a hydatid cyst (long arrows). Floating membrane within the dilated bile duct (short white arrows) and daughter cysts (asterisks) are also noted. Serological tests for *E*. *granulosus* were positive.

**Fig. 2.**
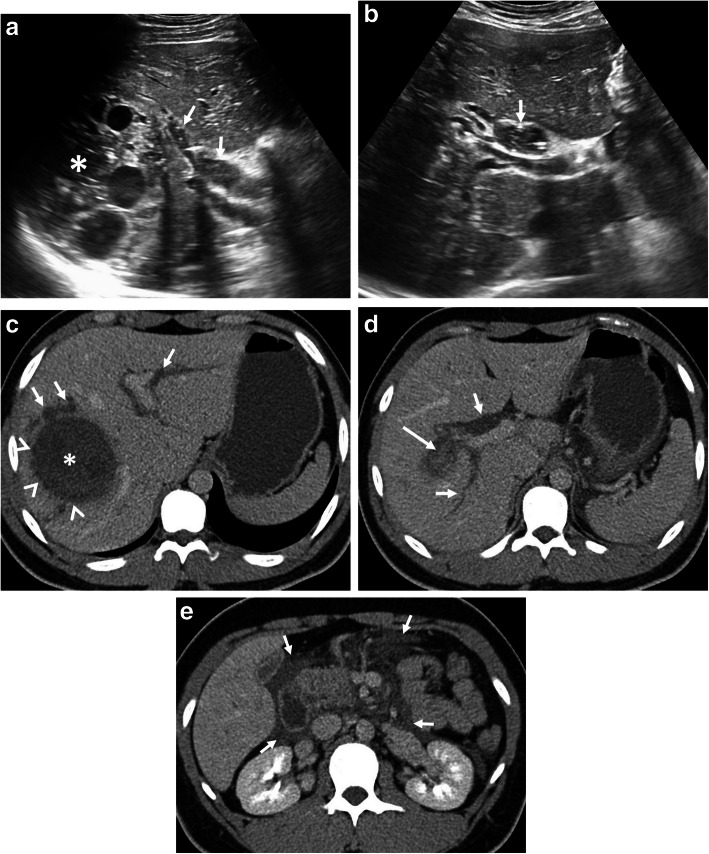
A 21-year-old man presented to the emergency department with a 1-week history of upper abdominal pain, pruritus, and jaundice. Laboratory tests revealed increased levels of serum amylase, lipase, and acute phase reactants. Serological tests for *E*. *granulosus* were positive. **a**, **b** Axial gray-scale US images demonstrate the imaging findings of intrabiliary rupture of a hydatid cyst (asterisk). The right main bile duct and choledochal duct are dilated due to cyst contents (arrows, **a**, and **b**). **c**–**e** Axial contrast-enhanced CT images reveal hydatid cyst (asterisk) and associated wall irregularity (arrowheads) due to intrabiliary rupture. Note biliary dilatation (short arrows, **c** and **d**) and also increased attenuation levels of the right main bile duct due to cyst contents (long arrow). CT image at the level of pancreas demonstrates peripancreatic fat tissue stranding (arrows, **e**) compatible with acute pancreatitis

**Fig. 3:**
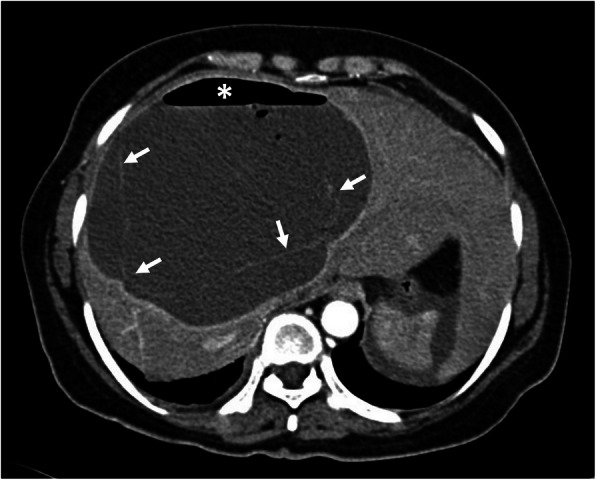
A 62-year-old woman presented to the emergency department with fever, fatigue, and right upper quadrant pain. The patient had a previous history of liver hydatid disease which was stable for 1-year of follow-up. Axial contrast-enhanced CT image shows a liver abscess. Floating membranes (arrows) and associated air-fluid level (asterisk) within the cyst indicating superinfection of a liver hydatid cyst. Culture test following percutaneous drainage of the cyst revealed Streptococcus anginosus infection

**Fig. 4:**
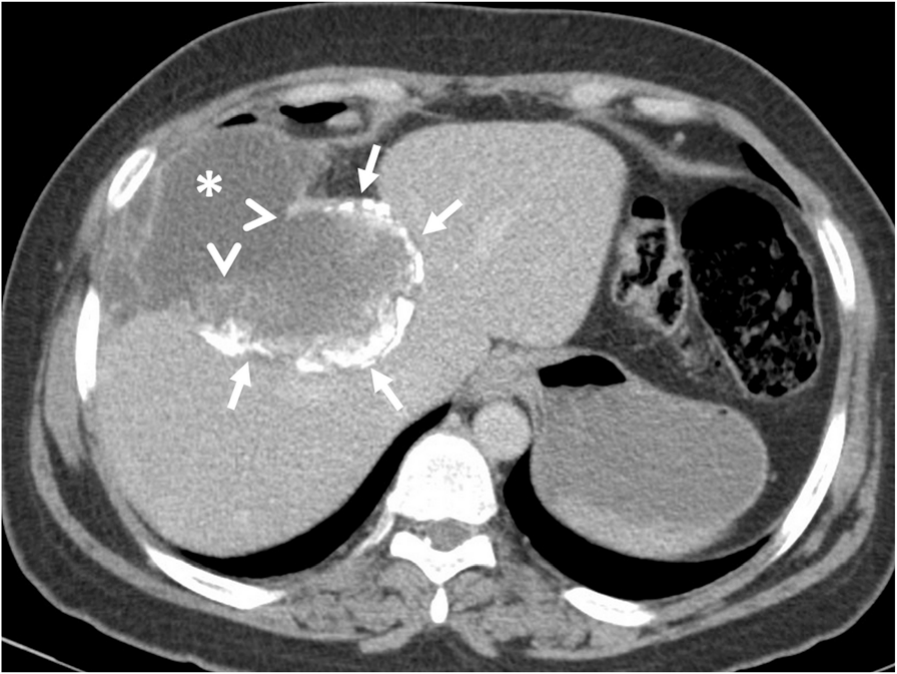
A 30-year-old woman was admitted to the emergency department with right upper quadrant pain. Axial contrast-enhanced CT image demonstrates calcified hydatid cyst (arrows) rupture through the perihepatic space. A fluid collection containing cyst content (asterisks) was found adjacent to the hepatic capsule due to the loss of integrity of the cyst wall (arrowheads). Surgical findings confirmed the rupture of the hydatid cyst into the perihepatic space

**Fig. 5:**
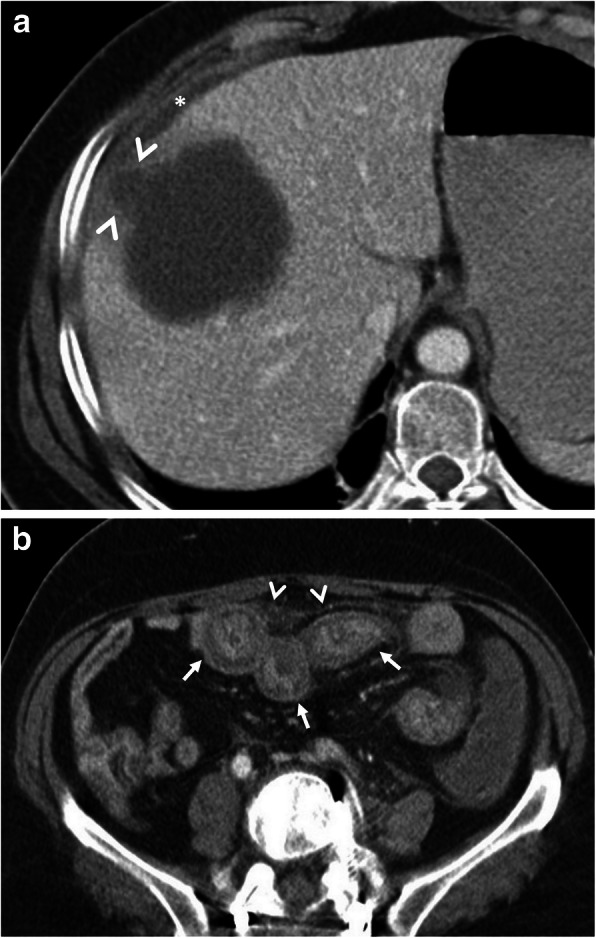
A 16-year-old girl was admitted to the emergency department with an acute onset of right upper quadrant pain and fever. **a** Axial contrast-enhanced CT image demonstrates the imaging findings of intraperitoneal rupture of a hydatid cyst. Focal outward bulging found at the lateral wall of the cyst indicates the site of rupture (arrowheads, **a**). Mild fluid collection adjacent to the hepatic capsule was also noted (asterisk). **b** Axial CT image from lower abdomen shows mural thickening of the jejunal loops (arrows) and adjacent fat tissue stranding (arrowheads) indicating the local allergic reaction of bowel wall secondary to ruptured hydatid cyst. Surgical findings confirmed the intraperitoneal rupture of the hydatid cyst

**Fig. 6:**
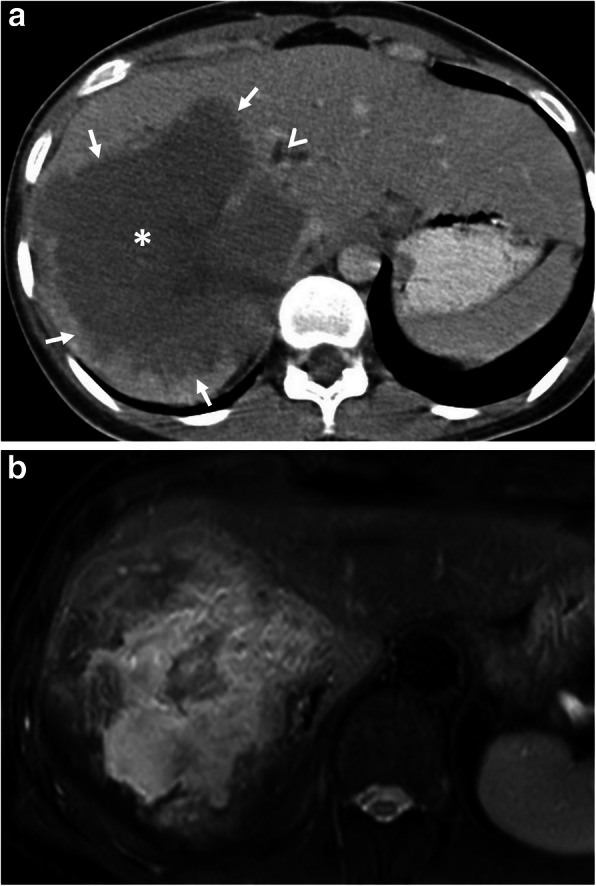
A 21-year-old woman was admitted to the emergency department with a 2-week history of jaundice and upper right quadrant pain. **a** Axial contrast-enhanced CT image demonstrates a heterogeneous infiltrative liver mass (asterisk) with irregular margins (arrows). The presence of biliary dilatation (arrowhead) due to compression of the mass was also noted. The diagnosis of alveolar echinococcosis was made by histopathological examination. **b** Fat saturated T2-weighted MR-image demonstrates internal heterogeneity of the mass mimicking primary liver malignancy. Chest CT findings were unremarkable for hydatid disease (not shown)

**Fig. 7:**
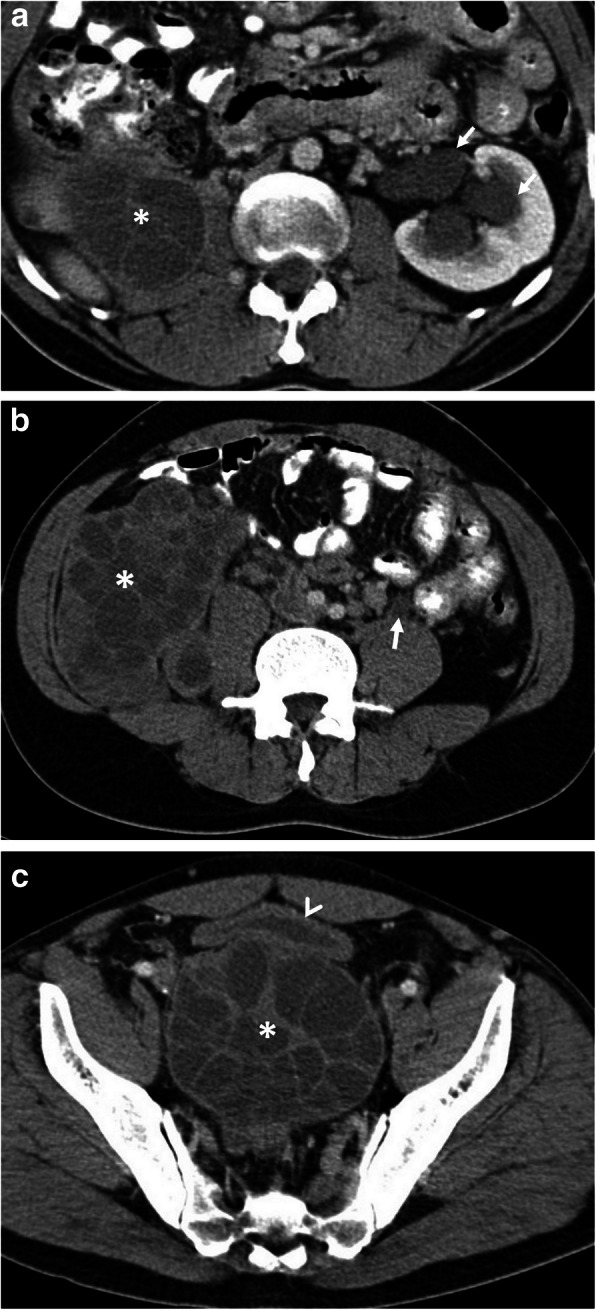
A 25-year-old man presented to the emergency department with a 1-week history of fever, voiding difficulty, and bilateral flank pain. Blood analysis revealed leukocytosis and elevated serum creatinine levels. **a**–**c** Axial contrast-enhanced CT images show a large retroperitoneal Gharbi type 3 hydatid cyst extending from the right kidney to the pelvic region (asterisks, **a**–**c**). The right kidney is no longer visible on CT due to the replacement of renal parenchyma with a hydatid cyst. Left-sided hydroureteronephrosis (arrows, **a** and **b**) was evident due to left ureteral compression by the pelvic portion of the hydatid cyst. Significantly compressed bladder (arrowhead, **c**) was also noted. Histopathological examination following surgery revealed the diagnosis of retroperitoneal hydatid disease and associated right kidney involvement

**Fig. 8:**
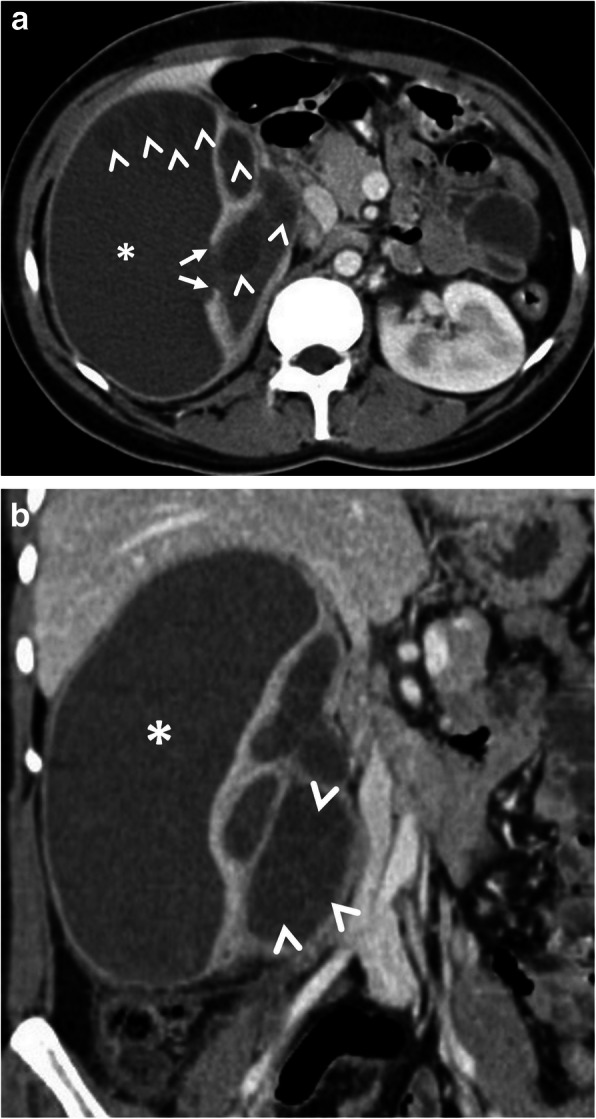
A 24-year-old woman was admitted to the emergency department with an acute onset of right flank pain and fever. Increased serum levels of acute-phase reactants, leukocytosis, and elevated serum creatinine levels were evident at blood analysis. **a**, **b** Axial (**a**) and coronal (**b**) contrast-enhanced CT images demonstrate a large renal subcapsular Gharbi type 3 hydatid cyst (asterisks) rupture into the pelvicalyceal system. Loss of integrity of the renal parenchyma found on CT image indicates the site of rupture (arrows, **a**). Daughter cysts within the cyst and also in the pelvicalyceal system are noted (arrowheads, **a**, **b**). The diagnosis was confirmed by histopathological examination following surgery

Hepatic hydatid cyst may cause acute abdominal pain due to its complications. The most common complication is the rupture and the most common site of the rupture is the biliary tree [21, 27, 28]. Other sites of the rupture are peritoneal cavity, thoracic cavity, hepatic subcapsular space, hollow viscera, and abdominal wall [21, 27]. The rupture may cause superinfection and anaphylaxis [27, 28]. The imaging findings of intrabiliary rupture of the hydatid cyst are structural deformity, loss of spherical shape, dilatation of the intrahepatic bile ducts, and linear filling defects within the biliary tract (Figs. 1 and 2) [21, 27, 28]. Also, air or an air-fluid level within the cyst may be present and this may indicate superinfection (Fig. 3) [28]. Intraperitoneal rupture is a rare complication and may cause peritoneal seeding (Fig. 4) [27, 29]. Superficial, large, and thin-walled hepatic hydatid cysts are most vulnerable to abdominal rupture [28]. The imaging findings of intraperitoneal rupture of the hydatid cyst are focal outward bulging, discontinuity of the cyst wall adjacent to the hepatic capsule, and intraperitoneal fluid collections [21, 27–29]. Also, mural thickening of the bowel loops and peritoneal fat tissue stranding may occur due to the local allergic reactions (Fig. 5). Large hydatid cysts in the liver or other organs may cause acute abdominal symptoms due to mass effect. Alveolar echinococcosis (*E*. *multilocularis*) may demonstrate an infiltrative growth pattern and patients’ symptoms are correlated with the size of the lesion. Compression of the biliary system may result in acute cholangitis and imaging findings may resemble those seen in liver malignancy (Fig. 6) [30]. Rupture or mass effect may be seen in other abdominal organ involvement (Fig. 7) [31]. In renal hydatid disease, the rupture of the hydatid cyst in the pelvicalyceal system is a rare complication (Fig. 8). Other rare complications of abdominal hydatid cyst (disease) are portal hypertension, portal vein thrombosis, and Budd-Chiari syndrome due to mass effect on the portal and hepatic veins [28].

## Fascioliasis

Fascioliasis is an important disease caused by *Fasciola hepatica* and *Fasciola gigantica*. *F*. *hepatica* is the most common type. Freshwater snail species are the intermediate hosts for fasciola parasites while many herbivorous mammals including humans are the definitive host [32]. *Fasciola* parasites develop into adult flukes in the bile ducts of infected mammals, which transfer immature *Fasciola* eggs through their feces [36].

The infection in humans begins when watercress or contaminated water containing encysted larva are ingested. The larvae reach the liver after penetrating the duodenal wall, migrating through the peritoneal cavity and penetrating the Glisson’s capsule. The flukes then migrate through the liver parenchyma to the bile ducts and gallbladder which are their permanent residence. The migratory larval and resting adult stages correspond to two clinical stages of the life cycle, called the hepatic and biliary stages [34, 35]. Common symptoms of the hepatic phase are urticaria, right upper quadrant pain, and fever. The biliary phase is usually accompanied by intermittent right upper quadrant pain, with or without cholangitis or cholestasis [36].

**Fig. 9:**
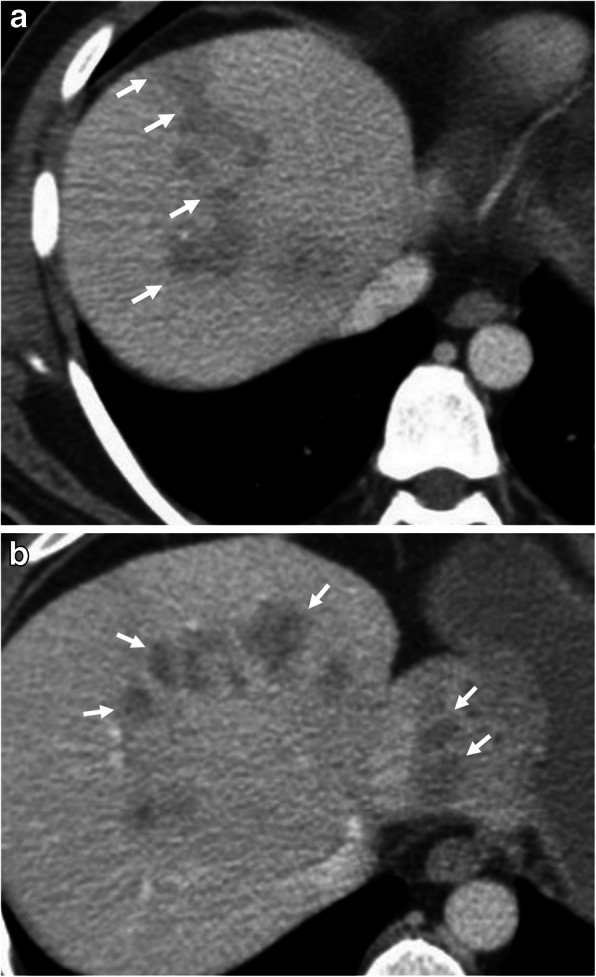
A 49-year-old man was admitted to the emergency department with a 1-week history of fatigue, fever, and abdominal pain. Blood analysis was unremarkable except for the presence of eosinophilia. **a**, **b** Axial contrast-enhanced CT-images demonstrate patchy areas of decreased attenuation migrating from liver capsule through to deep parenchyma (arrows, **a**, **b**). Although not confirmed histopathologically, imaging-based diagnosis of *F*. *hepatica* infection was made depending on the distribution of parenchymal hypodensities on CT

**Fig. 10:**
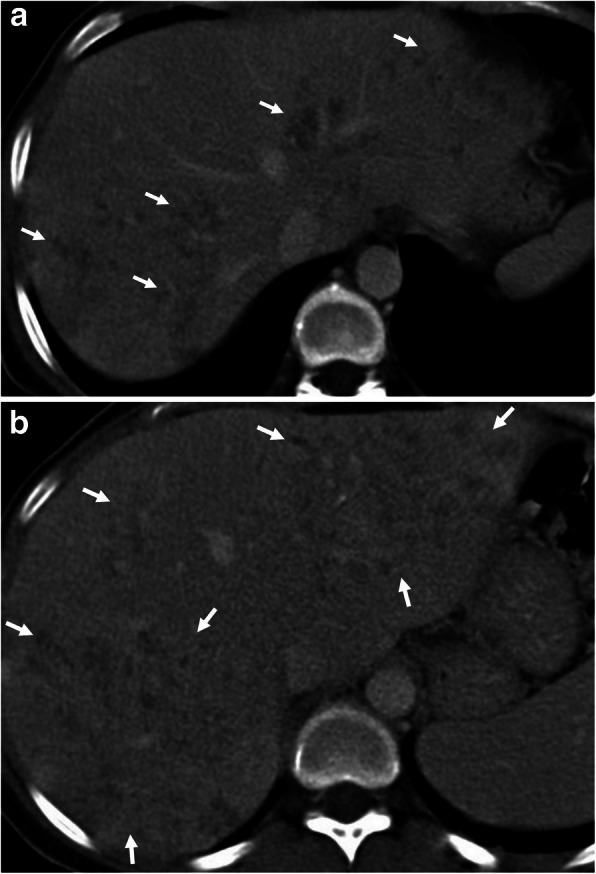
A 33-year-old woman was admitted to the emergency department with a 4-day history of right upper quadrant pain, fever, and fatigue. Blood analysis revealed leukocytosis, increased serum level of CRP, eosinophilia, and increased level of liver enzymes. **a**, **b** Axial contrast-enhanced CT-images show nodular irregular areas of decreased attenuation scattered through the liver parenchyma (arrows). The distribution of lesions was compatible with the clusters of microabscesses seen in *F*. *hepatica* infection. Indirect hemagglutination test positivity for *F*. *hepatica* and imaging findings supported the diagnosis

The migration process causes multiple microabscesses in the liver. The findings of the migration process become visible on CT as a “tunnel-like tract” from the entry site at the Glisson’s capsule deep to the parenchyma and clustered small necrotic cavities arranged in serpentine fashion are seen as “caves” [34–37]. “'Tunnels and caves” sign is very characteristic for fascioliasis (Figs. 9 and 10). Subcapsular hemorrhage-effusion, thickening, and enhancement of the focal liver capsule may occur due to penetration of the Glisson’s capsule by larva [36, 38]. Wedge-shaped hemorrhagic infarction in the periphery of the liver was reported as a result of an experimental study on hepatobiliary fascioliasis [39]. A case presenting portal vein thrombosis has also been reported [40]. Periportal lymphadenopathy is a frequent and helpful finding which can be encountered in both hepatic and biliary stage [38].

In the biliary stage, adult flukes in the extrahepatic bile ducts and gallbladder cause biliary epithelial hyperplasia, hypertrophy, and partially or complete biliary obstruction [34–36]. The obstruction causes acute abdominal pain due to complications such as cholestasis, cholecystitis, cholangitis, and pancreatitis [41–43]. Imaging features of acute abdominal pain due to biliary obstruction by adult flukes include dilated biliary ducts, linear echogenic floating particles in the biliary system on ultrasonography (US), and irregular thickening of bile duct walls, contrast enhancement of duct walls, and intermediate signal filling defects in the dilated ducts on T2-weighted MR images. Imaging features of accompanying microabscesses in the liver parenchyma such as heterogeneous echotexture due to focal small hypo- or hyperechoic lesions on US and round or oval clustered hypodense lesions with peripheral contrast enhancement on CT and MRI may be helpful in the differential diagnosis [34, 35, 38, 79]. A large cavitary lesion may occur rarely as a result of reinvasion of the hepatic parenchyma by an adult worm and rupture of the bile duct. As a rare complication of fascioliasis, acute hemobilia related to the bleeding ulcer in the bile duct may be observed [36].

## Ascariasis

*Ascaris lumbricoides* is the most common and the largest roundworm parasite of the human intestine [2]. *A*. *lumbricoides* are transmitted through the ingestion of contaminated food. After eggs are dissolved in the stomach to initiate larva formation, the larvae reach the caecum and migrate through the liver via penetrating portal vein branches. They reach hepatic veins via hepatic sinusoids and finally, the right heart and lungs are involved. They travel to bronchi and trachea and may be seen in sputum at this stage. When sputum is swallowed again, they reach the gastrointestinal system where they become adult worms [44].

**Fig. 11:**
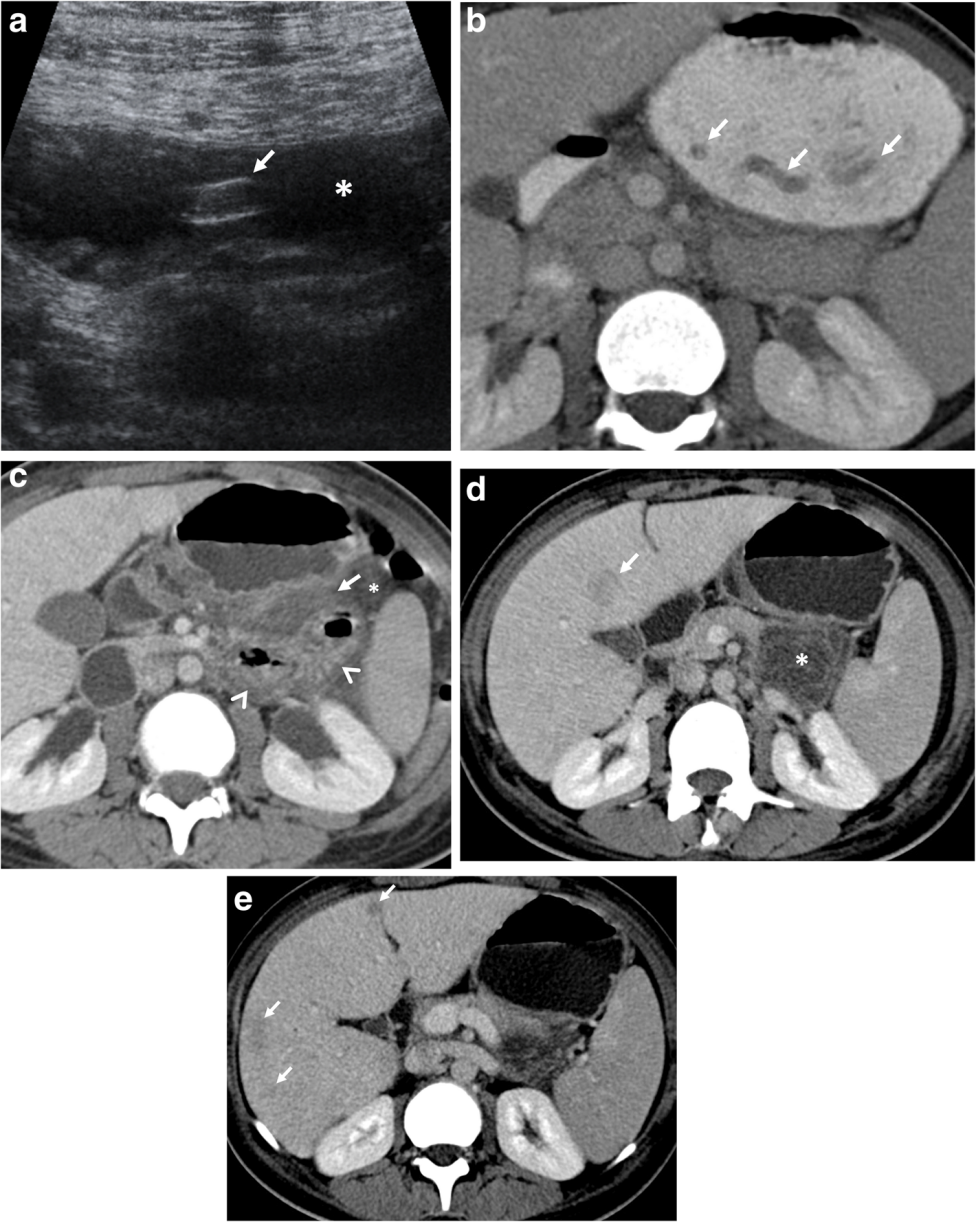
A 21-year-old woman was admitted to the emergency department with a 1-week history of vomiting and abdominal pain. Physical examination was unremarkable except for mild epigastric tenderness. Increased serum levels of acute-phase reactants and eosinophilia were evident at blood analysis. **a** Axial gray-scale US image demonstrates a tubular structure (arrow) within the lumen of the stomach (asterisk). **b** Axial contrast-enhanced CT scan more clearly depicts the worm (arrows). **c** Follow-up CT scan was obtained on the 3rd day of admission due to clinical suspicion of acute pancreatitis. Mild fat stranding (arrow) and fluid (asterisk) with jejunal wall thickening (arrowheads) were noted on CT. **d**, **e** Follow-up CT scans obtained on the 5th day of admission demonstrate the evolution of peripancreatic inflammatory changes into a heterogeneous fluid collection (asterisk, **d**). Peripherally located liver lesions (arrows, **d**, **e**) were also noted. Imaging findings raised suspicion for the involvement of visceral larva migrans. The definitive diagnosis was achieved with the identification of Ascaris eggs in the stool examination

Patients with ascariasis are asymptomatic or present with nonspecific abdominal symptoms. Acute abdominal pain may occur due to complications [2]. Small bowel obstruction is an expected complication in the massive infestation. Massive worm aggregates may cause bowel infarction and gangrene. The worms have a predilection for the orifices and the ampulla of Vater is frequently involved. Thus, biliary and pancreatic complications are also common [45]. Biliary colic, acute cholecystitis, recurrent cholangitis, liver abscess, and acute pancreatitis are related complications [45, 46]. Rarely the worm causes gastrointestinal perforation [47]. On sonography, the roundworm may be seen as single or multiple, long, linear, and thick echogenic strips without acoustic shadowing [48]. A central, longitudinal anechoic tube between two parallel echogenic lines, known as “triple line sign,” represents the digestive tract of the Ascaris worm [46, 49]. US may reveal the movement of the worm during real-time scanning. Acute intestinal obstruction secondary to ascariasis may present on CT as dilated bowel loops and elongated or rounded filling defects in the contrast filled lumen of the bowel [48]. Ascaris worms manifest as relatively hyperattenuating tubular structures surrounded by less attenuated bile in the setting of bile duct obstruction [48]. Acute pancreatitis caused by Ascaris worms presents on CT as diffusely dilated pancreatic duct and peripancreatic edema. (Fig. 11).

## Toxocariasis

**Fig. 12:**
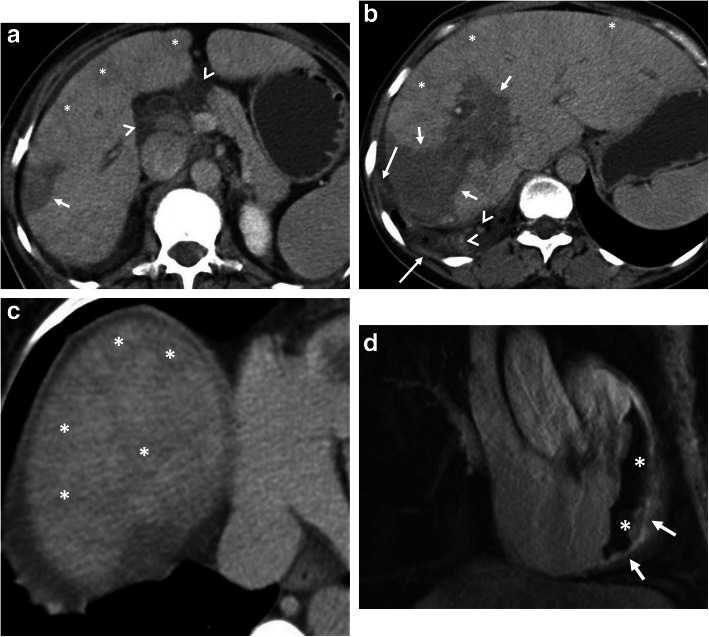
A 49-year-old woman was admitted to the emergency department with a 1-week history of fever, shortness of breath, and weight loss. Physical examination revealed tachycardia. Increased serum level of acute phase reactants, eosinophilia, and increased level of liver enzymes were evident at blood analysis. **a**–**c** Axial contrast-enhanced CT images demonstrate periportal fat tissue stranding (arrowheads, **a**) and parenchymal hypodensity affecting right liver lobe (arrows, **a**, **b**). Areas of decreased parenchymal attenuation scattered at the periphery of the liver were also noted on CT (asterisks, **a**–**c**). The presence of complex pleural effusion (black asterisks) containing nodular (arrowheads, **b**) and linear (long arrows, **b**) areas of contrast enhancement, was consistent with pleural involvement. Liver biopsy revealed liver injury and the presence of eosinophilic infiltration. CT image at the level of liver dome demonstrates the parenchymal involvement (asterisks, **c**). **d** Sagittal reformatted T1-weighted inversion-recovery MR image obtained 10 min following IV gadolinium-based contrast agent injection, shows biventricular apical thrombus (asterisks) and subendocardial contrast enhancement (arrows) suggestive of Loeffler’s endocarditis. The definitive diagnosis of the Toxocara infection was made by serological tests

Toxocariasis is the infection in the human host with *Toxocara canis* or *Toxocara cati*. Their definitive hosts are the domestic dog and cat. Humans (especially children) are accidentally infected hosts when they ingest the eggs from contaminated foods. The larvae hatch in the small intestine, penetrate the wall, and migrate to all organs via the bloodstream (visceral larva migrans) [80]. The liver, lungs, central nervous system, and eyes are the most affected organs. Cardiac involvement may rarely be encountered (Fig. 12). Tissue damage is largely dependent upon eosinophilic inflammation as a response of the host immune system to the dead larvae [80].

Clinically, most of the patients are asymptomatic. Heavy infection can cause fever, abdominal pain, general weakness, weight loss, and pulmonary and neurological symptoms [81, 82]. Liver involvement appears as small, oval, multiple hypoechoic lesions on US [81, 82]. On CT, the most common imaging finding of the hepatic toxocariasis is multiple, ill-defined, oval or sometimes angular or trapezoid in shape, low-attenuating nodules along the portal vein branches that are measured less than 2 cm, best seen on the portal venous phase. An enhancing rim may be seen on arterial phase images. When the infection becomes severe, the small lesions fuse to form large lesions, some of which are crossed by portal vein branches (Fig. 12) [81, 82]. Toxocariasis appears as single or multiple lesions with low signal intensity on T1-weighted images and high signal intensity on T2-weighted images. Portal phase images after intravenous (IV) gadolinium administration are the most helpful method to demonstrate poorly defined low-signal intensity lesions. Concomitant lung lesions may also be seen in severe infection [82]. Cardiac involvement including myocarditis, pericarditis, and Loeffler’s endocarditis may be encountered in rare cases with Toxocariasis [83].

## Intestinal taeniasis

Human taeniasis is a parasitic infection caused by three tapeworm species, *Taenia saginata*, *Taenia solium*, and *Taenia asiatica*. Humans are the only definitive hosts for these *Taenia* tapeworms. Humans become infected with tapeworms when they eat raw or undercooked beef or pork containing infective cysticerci [50]. Intestinal taeniasis is acquired by ingesting *T*. *solium* or *T*.*saginata* larvae, whereas ingestion of *T*. *solium* eggs causes cysticercosis [50]. Following *Taenia solium* ingestion, the embryos may penetrate the bowel wall and enter portal circulation. Afterward, they reach distal capillaries (cysticercosis) of richly perfused tissues (central nervous system, skeletal muscle, eyes, and subcutaneous tissue) [18, 50].

**Fig. 13:**
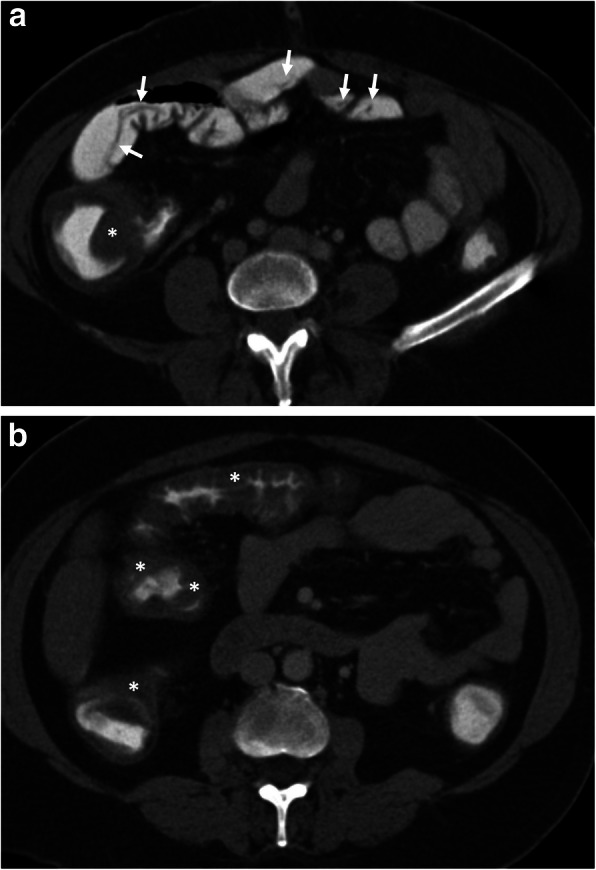
A 45-year-old woman was admitted to the emergency department with a 2-week history of diarrhea and abdominal pain. **a**, **b** Axial contrast-enhanced CT images demonstrate multiple tubular filling defects in the small bowel (arrows). Colonic wall thickening, indicating colitis, is also noted (asterisks, **a**, **b**). The definitive diagnosis of intestinal taeniasis was achieved following the stool examination

Most people with tapeworm infections have no symptoms or mild symptoms. But rare complications such as intestinal obstruction, intestinal perforation, gallbladder perforation, acute pancreatitis, acute appendicitis, and acute cholecystitis may cause acute abdominal pain [51–55]. Adult worms in gallbladder lumen, bile ducts, and pancreatic duct become visible as linear hyperechoic materials on US with dilatation of the involved ducts [53]. Intestinal taeniasis appears as a fine, tubular structure with hyperechoic walls in the intestinal lumen on US (Fig. 15). Taenia worms cause double-reflective, ribbon-like echo in the lumen of the bowels (Fig. 13) [56]. MRCP images demonstrate hypointense linear structures within the dilated intra- and extrahepatic bile ducts in biliary system involvement [59]. The presence of abundant free fluid and free air on imaging studies suggests perforation of luminal organs involved by the parasite [51, 52].

## Amoebiasis

Amoebiasis is a parasitic disease caused by *Entamoeba histolytica*. Humans are the only natural hosts. Ingestion of the cyst from contaminated food or water initiates the infection. The cyst forms the trophozoite stage within the terminal ileum or colon [57]. The trophozoite form causes disease and may spread to the extraintestinal localizations via the portal vein, the lymphatics, or by direct extension through the peritoneum [58].

**Fig. 14:**
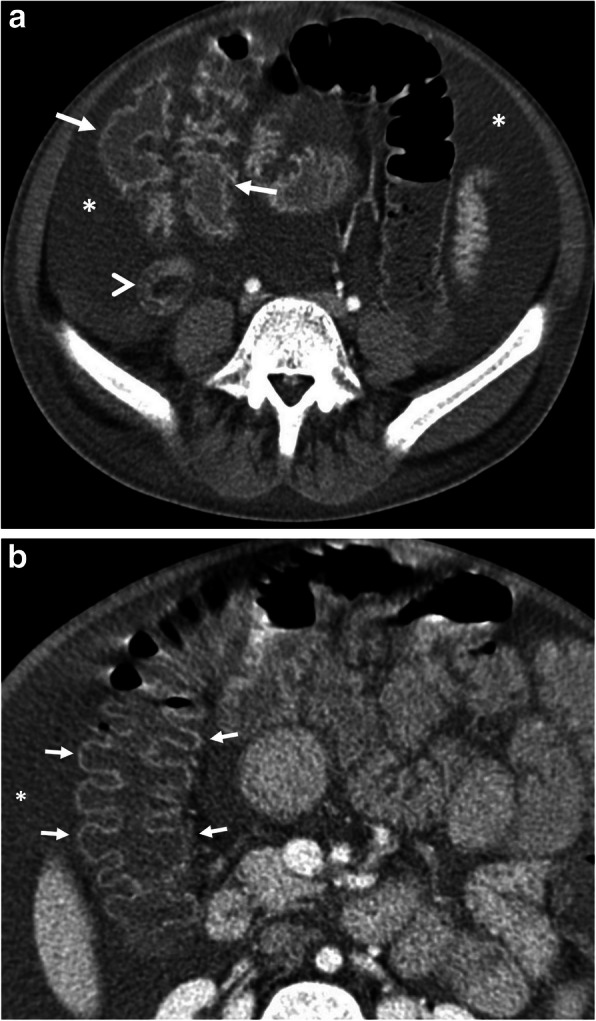
A 12-year-old boy was admitted to the emergency department with a 3-day history of hematochezia and diarrhea. He also complained of abdominal pain and abdominal distention. Physical examination revealed diffuse abdominal tenderness. Increased serum levels of acute-phase reactants and leukocytosis were evident at blood analysis. **a**, **b** Axial contrast-enhanced CT images demonstrate wall thickening and contrast enhancement of the appendix vermiformis (arrowhead) and ileum (arrows, **a**). Intraabdominal free fluid (asterisks, **a**, **b**) and increased mucosal enhancement of the colon (arrows, **b**) are also noted. Mucosal ulcers were evident at colonoscopy. Histopathological examination revealed the presence of trophozoits of *Entamoeba histolytica*, superficial ulcers, and lymphangiectasia at the lamina propria of the small bowel

Most of the infected patients are asymptomatic; however, about 10% may show with clinical symptoms. The right-side colon tends to be more severely involved. The terminal ileum may be involved up to 10% [44]. Acute amebic colitis presents with bloody diarrhea, tenesmus, and abdominal pain [57, 58]. In amebic colitis, CT may demonstrate wall thickening, mucosal edema, and increased mucosal enhancement (Fig. 14) [44]. Fulminant amebic colitis is characterized by transmural extension of amebic colitis which may result in total colonic gangrene and bowel perforation [44, 59]. Imaging features of fulminant amebic colitis include deep ulcerations, submucosal and intramural tracking of contrast agent, and discontinuous bowel necrosis seen as alternating enhancing and nonenhancing bowel wall [84].

The most common extraintestinal manifestation of the disease is a liver abscess. Symptoms of liver abscess are fever, right upper quadrant pain, and tenderness [57]. Amebic abscesses are predominantly solitary, oval or round shaped, and located near the liver capsule in the right lobe of the liver. Sonographic features of amebic abscess consist of a hypoechoic lesion with low-level internal echoes and the absence of significant wall echoes. On CT scan, it is seen as a low-density lesion with a peripheral enhancing rim thickened wall. A mild contrast enhancement around the abscess in the adjacent liver parenchyma occurs due to the peripheral zone of edema [85]. Amebic liver abscess is frequently together with right-sided pleural effusion and perihepatic fluid [44].

## Malaria

Malaria is a serious parasitic infectious disease that is caused by *Plasmodium* species. Plasmodium species are transmitted through infected female mosquitoes that bite humans. These parasites grow within erythrocytes and are released by cyclic hemolysis [60].

**Fig. 15:**
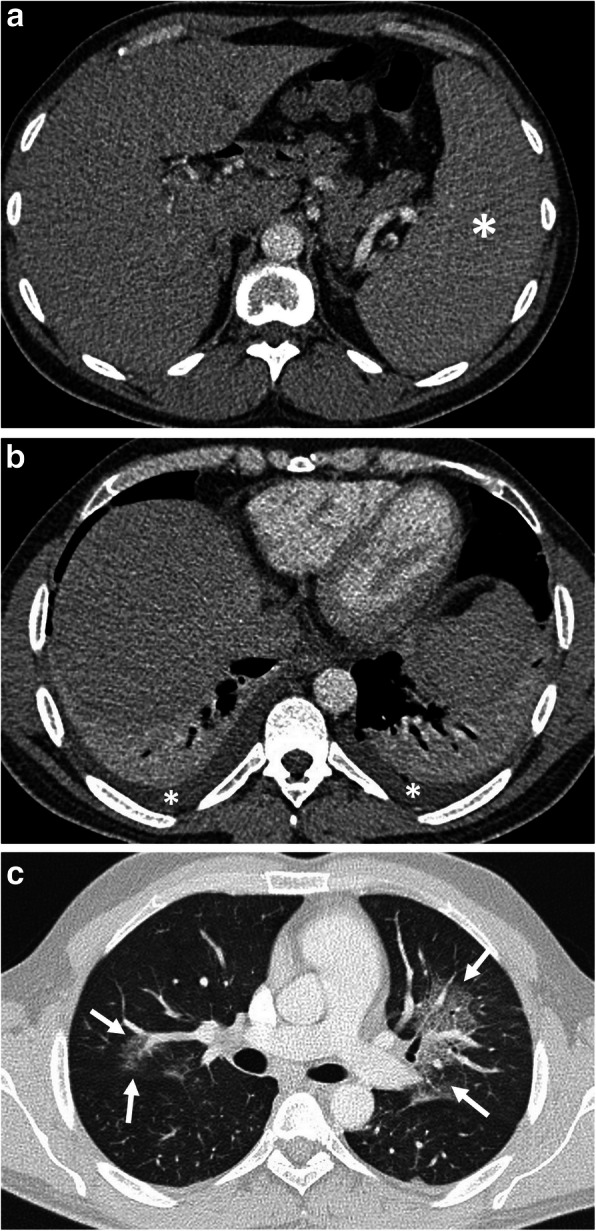
A 39-year-old man was admitted to the emergency department with a 1-week history of mild abdominal pain and fever. Physical examination was unremarkable except for splenomegaly. Blood analysis demonstrated thrombocytopenia. **a** Axial contrast-enhanced CT image shows splenomegaly (asterisk). There was no sign of splenic infarction, hematoma, and rupture at the time of diagnosis. **b**, **c** CT scan of the chest showed bilateral mild pleural effusion (asterisks, **b**) and centrally located ground-glass opacities (arrows, **c**). The findings raised suspicion for non-cardiogenic pulmonary edema. The imaging findings were not specific for any particular type of disorder. Nevertheless, the final diagnosis was made by a peripheral blood smear examination showing the plasmodium falciparum infection

The main symptom of malaria is episodic fever [60]. One of the most commonly involved systems during acute malaria is the gastrointestinal tract. Gastrointestinal symptoms are common in children with falciparum malaria. Abdominal symptoms of malaria are vomiting, dyspepsia, diarrhea, abdominal pain, and intestinal hemorrhage [61]. Acute pancreatitis, acalculous cholecystitis, and subacute intestinal obstruction are rare complications of the falciparum malaria. The precise mechanism of these complications is not clearly defined. *Plasmodium falciparum* infection was stipulated to have resulted in microvascular obstruction and consequently result in affected organ ischemia [14]. Rarely perforation can also occur [62]. Splenomegaly is the most common finding on CT examination in patients with gastrointestinal symptoms (Fig. 15). Other findings are hepatomegaly, splenic infarction, spontaneous splenic rupture and hemorrhage, ascites, and periportal edema [60, 63]. In patients with malaria and acute abdominal pain, CT is the mainstay imaging technique in the differentiation between splenic infarction and spontaneous rupture. Discontinuation of splenic contours, heterogeneous enhancement of splenic parenchyma, and perisplenic and intraabdominal free fluid with density levels ranging between 35 and 60 HU that suggests hemoperitoneum and presence of highest attenuation of the intraabdominal free fluid (sentinel clot) nearest to spleen should suggest splenic rupture in patients with suspicion of malaria due to travel history to the endemic areas [64]. Patients with malaria have also been reported to develop non-cardiogenic pulmonary edema and acute respiratory distress syndrome (ARDS) due to increased capillary permeability or endothelial damage (Fig. 15) [65, 66].

## Visceral leishmaniasis

Leishmaniasis is a vector-borne parasitic disease caused by *Leishmania* species. There are 3 main forms of leishmaniases: cutaneous, mucocutaneous, and visceral [68]. Persistent irregular fever and splenomegaly are characteristic findings for visceral leishmaniasis. Besides, lymphadenopathy, hepatomegaly, pallor, night sweats, weakness, anorexia, asthenia, cutaneous pigmentation, and weight loss may be seen. Left upper quadrant pain may be caused by massive splenomegaly [68]. Gastrointestinal hemorrhage may be encountered in leishmaniasis due to the involvement of bowels as the duodenum being the most frequently involved segment [69].

**Fig. 16:**
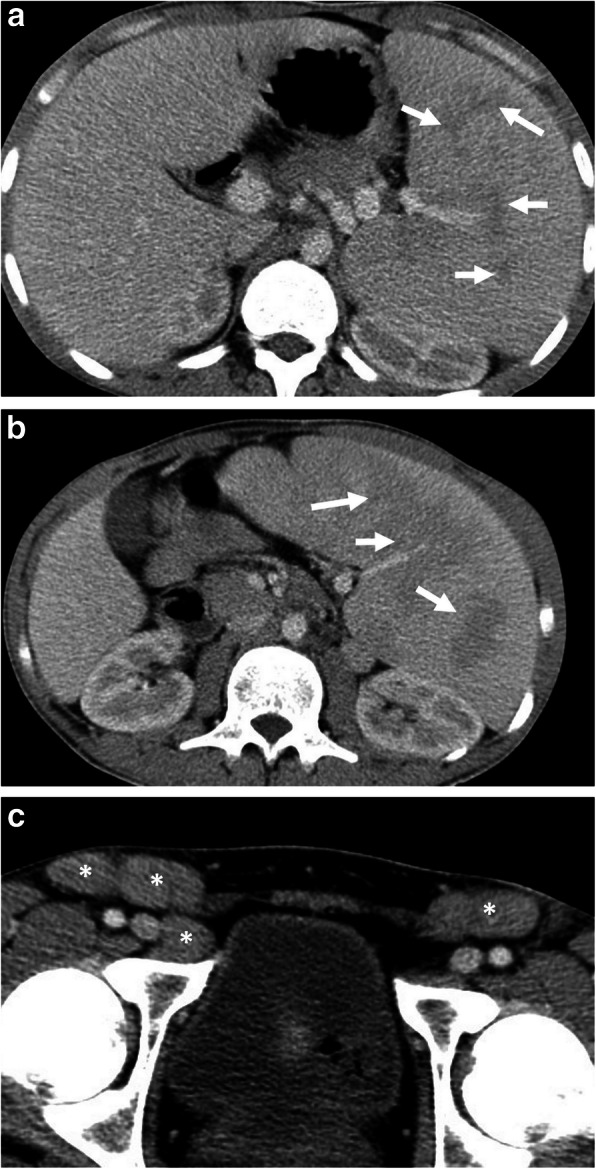
A 21-old man was admitted to the emergency department with a 2-week history of left upper quadrant abdominal pain and fever. Physical examination revealed splenomegaly and inguinal lymphadenopathy. Increased serum levels of acute-phase reactants, leukocytosis, and high sedimentation were evident at blood analysis. **a**, **c** Axial contrast-enhanced portal venous phase CT images demonstrate patchy areas of hypoattenuation (arrows, **a**, **b**) within the enlarged spleen and bilateral inguinal lymphadenopathy (asterisks, **c**). The liver was normal except for mild hepatomegaly (18 cm). The diagnosis of Leishmaniasis was made by histopathological examination following splenectomy

Regarding US findings in the patients, splenomegaly, lymphadenopathy, and hepatomegaly are common. Less common sonographic findings include ascites, pleural effusion, portal vein dilatation, and inferior vena cava dilatation. Increased echogenicity of the kidneys and decreased echogenicity of the pancreas may be seen very rarely [70]. On contrast-enhanced CT, hepatosplenomegaly, hypodense nodular lesions in the liver, and spleen and lymphadenopathy may be detected (Fig. 16). CT angiography may reveal intraluminal contrast extravasation in cases with gastrointestinal hemorrhage. In the differential diagnosis, lymphoma should be kept in mind due to the above-mentioned mimicking findings [71, 72]. Rarely, splenic infarction and splenic rupture may occur.

## Anisakiasis

Ingestion of raw or undercooked fish or other seafood may cause a rare parasitic disease, Anisakiasis [75]. There is a controversy in previous reports about the ability of the larvae of genus Anisakis during penetration of the gastrointestinal wall. Some reports indicate that the larva can only be stuck on the gastrointestinal wall while others emphasize the penetrating ability of the parasite [75–77]. Attachment of larvae in the gastrointestinal wall causes local tissue damage including ulceration, granulomatous inflammation, or perforation while an allergic reaction of the gastrointestinal wall or IgE-mediated systemic allergic reaction may also occur [76]. Gastric involvement forms 90–95% of cases [77]. Symptoms include abrupt onset of abdominal pain, nausea, vomiting, fever in gastric anisakiasis with additional diarrhea, peritoneal irritation, and intestinal obstruction caused by intussusception in intestinal anisakiasis [75, 77]. Diagnosis of anisakiasis with imaging findings solely is usually difficult if one neglects the history of raw or undercooked seafood consumption. US, as a frequently used imaging technique in epigastric or right upper quadrant pain, may demonstrate diffuse concentric wall thickening in gastric or intestinal walls with hypoechoic submucosal edema, irregularity of the lumen surface due to edema of the Kerckring’s folds (corn sign), and accompanying free fluid around involved segment [75].

The features of anisakiasis in CT imaging during gastric and intestinal involvement include gastric or intestinal wall thickening due to submucosal edema, perigastric fat stranding in gastric anisakiasis, and trace ascites. Prompt diagnosis of gastric anisakiasis can be accomplished with endoscopy followed by endoscopic removal of larvae. Management of intestinal anisakiasis depends on conservative medical treatment with antihelminthic drugs such as albendazole [77].

## Dientamoebiasis

**Fig. 17:**
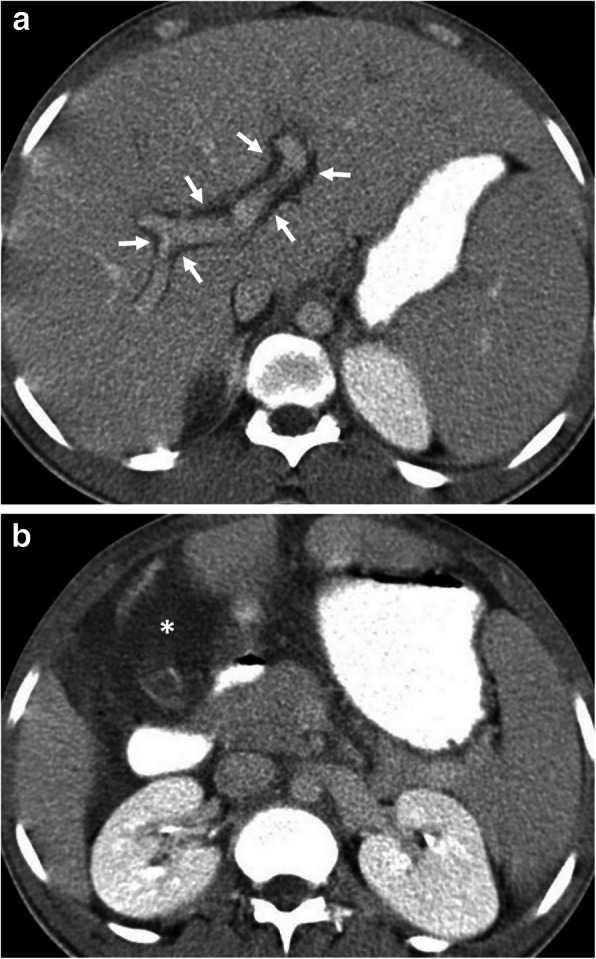
A 23-year-old man was admitted to the emergency department with a 1-week history of abdominal pain and diarrhea. Physical examination and blood analysis were unremarkable except for mild abdominal tenderness and eosinophilia, respectively. **a**, **b** Axial contrast-enhanced CT images demonstrate the presence of intra-abdominal free fluid (asterisk) and periportal edema (arrows). The imaging findings did not raise suspicion for an individual pathology. There was no definitive proof that this organism was the cause of the symptoms or imaging manifestation. Nevertheless, the patient’s symptoms and imaging findings were completely resolved following the treatment for *Dientamoeba fragilis* which was found on the patient’s stool examination

*Dientamoeba fragilis* is a protozoan parasite of the human bowel. It has been considered for years to be a nonpathogenic organism but more recent reports throughout the world show association of this parasite with gastrointestinal symptoms [86]. The most common symptoms of dientamoebiasis are diarrhea, abdominal pain, loose stools, and anorexia [87]. Acute abdominal pain is a rare symptom [73, 88]. CT may demonstrate nonspecific inflammatory changes due to involvement (Fig. 17).

## Conclusion

Acute abdominal pain may be seen in parasitic diseases. The diagnosis can be delayed because of the similar clinical signs encountered in other frequently seen causes of acute abdomen. Therefore, parasitic infections should be included in the differential diagnosis of acute abdominal pain particularly in patients from known endemic areas. Awareness of imaging findings in abdominal parasitic involvement is triggering to raise the suspicion for parasitic disease as a cause of acute abdominal pain.

## Data Availability

Data sharing is not applicable to this article as no datasets were generated or analysed during the current study.

## Abbreviations

CT: Computed tomography
MRI: Magnetic resonance imaging
US: Ultrasonography
